# Non-noble metal-catalyzed cross-dehydrogenation coupling (CDC) involving ether α-C(sp^3^)–H to construct C–C bonds

**DOI:** 10.3762/bjoc.19.94

**Published:** 2023-09-06

**Authors:** Hui Yu, Feng Xu

**Affiliations:** 1 Department of Pharmacy, Shi zhen College of Guizhou University of Traditional Chinese Medicine, Guiyang, Guizhou 550200, P. R. China; 2 School of Mathematics and Information Science, Guiyang University, Guiyang, Guizhou 550005, P. R. China

**Keywords:** alkylation, cross-dehydrogenation coupling, ether, non-noble metals

## Abstract

Ether derivatives are widespread as essential building blocks in various drugs, natural products, agrochemicals, and materials. Modern economy requires developing green strategies with improved efficiency and reduction of waste. Due to its atom and step-economy, the cross-dehydrogenative coupling (CDC) reaction has become a major strategy for ether functionalization. This review covers C–H/C–H cross-coupling reactions of ether derivatives with various C–H bond substrates via non-noble metal catalysts (Fe, Cu, Co, Mn, Ni, Zn, Y, Sc, In, Ag). We discuss advances achieved in these CDC reactions and hope to attract interest in developing novel methodologies in this field of organic chemistry.

## Introduction

**Scheme 1:**
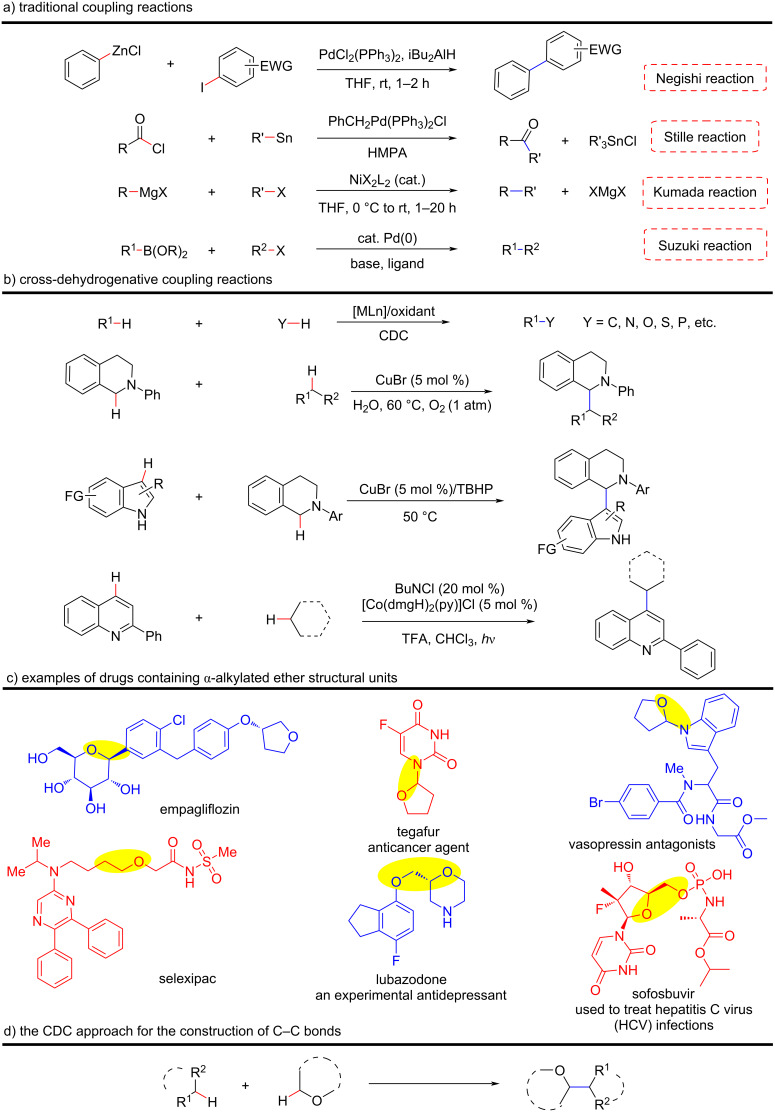
Research progress of coupling reactions and active compounds containing α-C(sp^3^)-functionalized ethers.

Since the 1970s, organic chemists have developed many selective cross-coupling methods for the construction of C–C bonds, such as the Negishi reaction (Zn) [1], Stille reaction (Sn) [2], Kumada reaction (Mg) [3], and Suzuki reaction (Pd) [4] (Scheme 1a). However, these coupling reactions involve a metal exchange step that generates a considerable amount of reaction waste, such as metal salts, which are not environmentally friendly. To overcome the shortcomings of the above coupling reactions, organic chemists have envisaged the construction of C–C bonds directly through C–H bond activation [5]. Fortunately, scientists have used various transition metals as catalysts to realize the activation of various types of C–H bonds, and have achieved fruitful scientific research results [6–12]. However, many of these coupling reactions usually require a prefunctionalization of the substrate, which makes the reaction sequences lengthy and inconsistent with the principle of atomic economy, thereby limiting broad application and further development.

To avoid the prefunctionalization of substrates, Li et al. first pioneered the concept of direct cross-dehydrogenative coupling (CDC) through continuous exploration and discovery [7,13–15]. The CDC reaction has flourished due to its versatility and has become an important synthetic strategy for the construction of C–C bonds in organic synthesis (Scheme 1b). Compared with traditional coupling reactions, the CDC strategy has the following advantages: 1) atom economy, 2) green and efficient reaction, 3) a wide range of substrate sources. Therefore, organic chemists consider the CDC reaction to be a new generation method for the construction of C–C bonds and it has received extensive attention and in-depth research [16–24].

The building blocks of ethers are widely found in biomass, chemical feedstocks, biologically active drugs, and natural products [25–31]. Ether motifs can significantly modify the pharmacological properties of parent molecules, including increasing lipophilicity and affecting half-life, which play a crucial role in biology, pharmaceuticals, and pesticides [32–33]. Some examples of ether-containing drugs are shown in Scheme 1c: Empagliflozin to treat type-2 diabetes [34–36], tegafur is used in medicine to treat a variety of cancers [37–38], lubazodone is an experimental antidepressant [39–40], and sofosbuvir is used to treat hepatitis C virus (HCV) infections [41–42]. For the synthesis of drug and natural compounds containing functionalized ether α-C(sp^3^)–H bonds CDC reactions can be applied. This review mainly focuses on the CDC reactions of ether oxygen α-C(sp^3^)–H bonds via non-noble metal-catalysis (Scheme 1d).

## Review

### Non-noble metal-catalyzed CDC reactions involving ether α-C(sp^3^)–H bonds

**Scheme 2:**
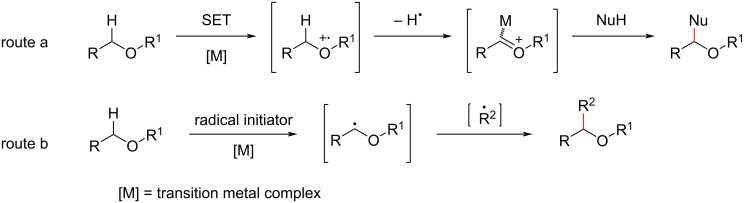
Transition-metal-catalyzed CDC pathways.

The possible mechanism of the CDC reaction involving ether α-C(sp^3^)–H bonds mainly follows the two pathways outlined in Scheme 2. Route a: First, the C(sp^3^)–H bond at the α-position of the oxygen atom undergoes a single-electron transfer under the combined action of the transition metal and an oxidant to generate an oxygen-radical cationic intermediate, which undergoes abstraction of a hydrogen radical (or loses a proton first, followed by an electron) to afford an oxonium ion intermediate. Finally, the oxonium ion is attacked by various nucleophiles to obtain the target functionalized product. Route b: the α-C(sp^3^)–H bonds are activated by a combination of transition metals and radical initiators to give the alkyl radicals, which are coupled with other radical receptors to afford the target product.

#### Cu-catalyzed reactions

Copper (common oxidation states are +I, +II and +III) has a significantly different reactivity and chemical selectivity from noble metals (Ru, Rh, Pd). Compared with noble metals, copper catalysts are cheaper and easier to obtain, making Cu more advantageous for industrial applications of C–H functionalization reactions. The Glaser–Hay reaction may be one of the oldest Cu-catalyzed oxidative coupling reactions [43]. However, due to complex mechanisms, Cu-catalyzed C–H functionalization reactions developed only slowly in the last decade. Since recently the Cu-catalyzed oxidative coupling has emerged as a powerful synthetic strategy due to the development of CDC reactions. Although the range of substrates for different C–H nucleophiles remains restricted, in recent years, copper-catalyzed oxidative coupling reactions between different C–H nucleophiles have been established. There are several common valence changes of copper in the catalytic process [44–50]: 1) Cu^II^ → Cu^I^ → Cu^II^; 2) Cu^I^ → Cu^III^ → Cu^I^; 3) Cu^II^ → Cu^III^ → Cu^I^ → Cu^II^.

**Scheme 3:**
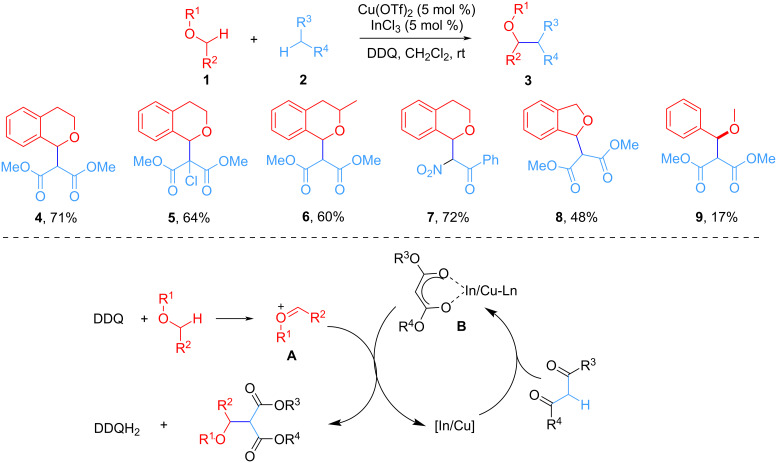
CDC of active methylene compounds in the α-C(sp^3^) position of ethers.

In 2006, Li et al. demonstrated that the CDC reaction of the C(sp^3^)–H bond of malonate diesters or other active methylene compounds with the C(sp^3^)–H bond adjacent to the oxygen atom of cyclic and open-chain benzylic ethers occurs at room temperature in the presence of Cu(OTf)_2_/InCl_3_ as catalysts and DDQ as oxidant (Scheme 3) [51]. By this route, a series of 2-alkoxymalonate diester derivatives was synthesized through direct CDC reaction. The mechanism study showed that the first step of the catalytic cycle involves a hydride abstraction from the benzylic site of isochroman to generate a cationic species **A**, whereas the malonate is activated by the In/Cu catalyst (**B**). Subsequently, the coupling of the two intermediates yields the desired product and regenerates the catalyst. Alternatively, In(III) may be involved in the activation of DDQ by coordinating the carbonyl oxygen atom which leads to an increase in the oxidation activity of DDQ.

**Scheme 4:**
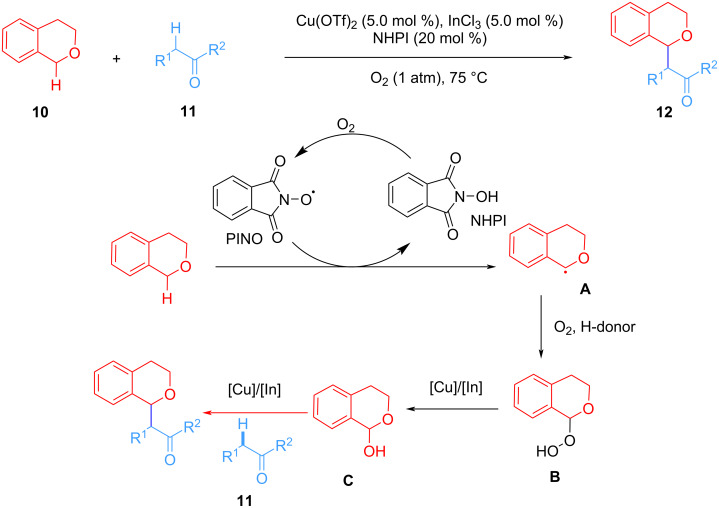
InCl_3_/Cu(OTf)_2_/NHPI co-catalyzed CDC reaction.

Subsequently, Li et al. improved the above method, using a mixture of indium and copper salts as a catalyst, NHPI (*N*-hydroxyphthalimide) as a co-catalyst to achieve the oxidative alkylation of cyclic benzyl ethers with malonates or ketones. Oxygen is used as a terminal oxidant at atmospheric pressure. The key intermediate of this oxidative coupling reaction is benzyl alcohol intermediate **C** (Scheme 4) [52]. The generation of N–O radicals from NHPI in the presence of oxygen triggers the whole coupling reaction. The potential application of NHIP as a catalyst for oxidative coupling reactions with oxygen as a terminal oxidant was explored.

**Scheme 5:**
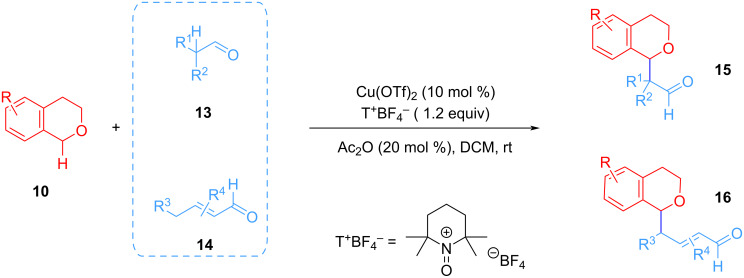
CDC of cyclic benzyl ethers with aldehydes.

In 2011, Garcia-Mancheño et al. developed a Cu-catalyzed CDC of cyclic benzyl ethers **10** with aliphatic or α,β-unsaturated aldehydes **13** or **14** through double C(sp^3^)–H/C(sp^3^)–H functionalization using 2,2,6,6-tetramethyl-*N*-oxopiperidin-1-ium tetrafluoroborate (T^+^BF_4_^−^) salt as the oxidant (Scheme 5) [53]. A catalytic amount of Ac_2_O played a significant role in the reaction, which can significantly improve the yield and selectivity of the reaction.

**Scheme 6:**
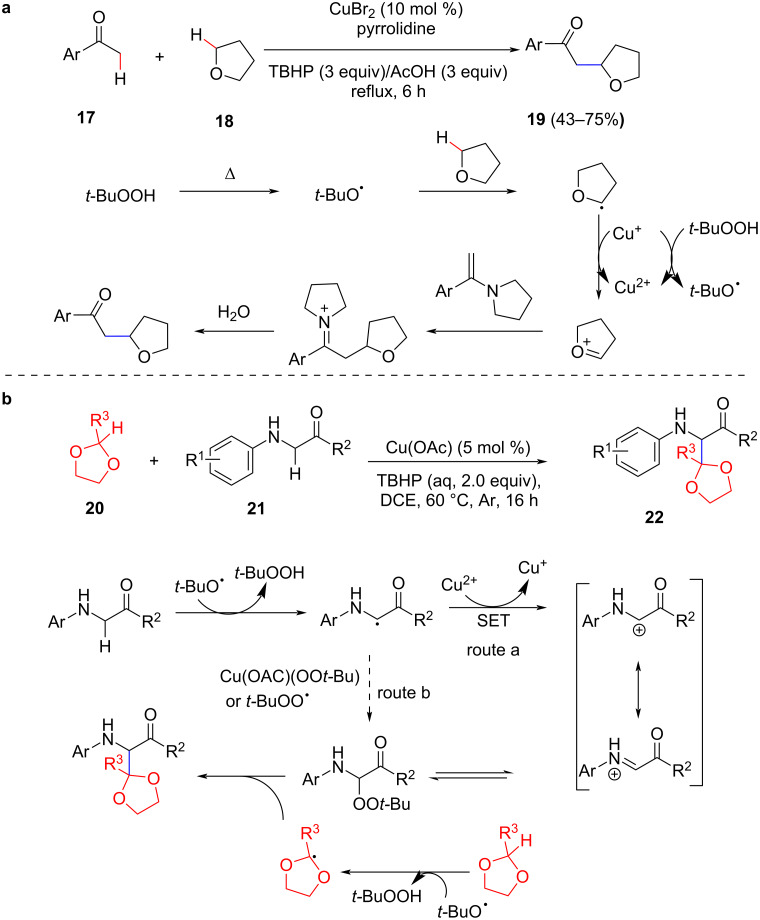
Cu-catalyzed CDC of (a) unactivated C(sp^3^)–H ethers with simple ketones and (b) double C(sp^3^)−H functionalization reaction of α-aminocarbonyl compounds with 2-alkyl-1,3-dioxolanes.

Due to the challenges associated with the activation of C(sp^3^)–H bonds, this kind of activation strategy has received extensive attention. Huang et al. developed a Cu-catalyzed CDC of unactivated C(sp^3^)–H ethers with simple ketones under the synergistic effect of CuBr_2_ and pyrrolidine. By this route, tetrahydrofuran or tetrahydropyran can smoothly react with many methyl aryl ketones to obtain the desired coupling products (Scheme 6a) [54]. The mechanism of the dehydrogenation cross-coupling reaction may undergo a radical pathway. Initially, the *tert*-butoxy radical produced by the dissociation of *t*-BuOOH may extract a hydrogen from the ether C (sp^3^)–H bond to form radicals. Subsequently, a single electron transfer (SET) leads to the oxonium species. Then, the enamine generated in situ from methyl aryl ketone and pyrrolidine undergoes a nucleophilic reaction with the oxonium species followed by hydrolysis to form the coupling product. However, this method is only applicable to cyclic ethers. In the same year, Correa et al. established a double C(sp^3^)–H functionalization reaction of α-amino carbonyl compounds and 2-alkyl-1,3-dioxolanes in the presence of Cu(I) (Scheme 6b) [55]. This method allows the synthesis of compounds with quaternary centers and natural products with high structural complexity.

**Scheme 7:**
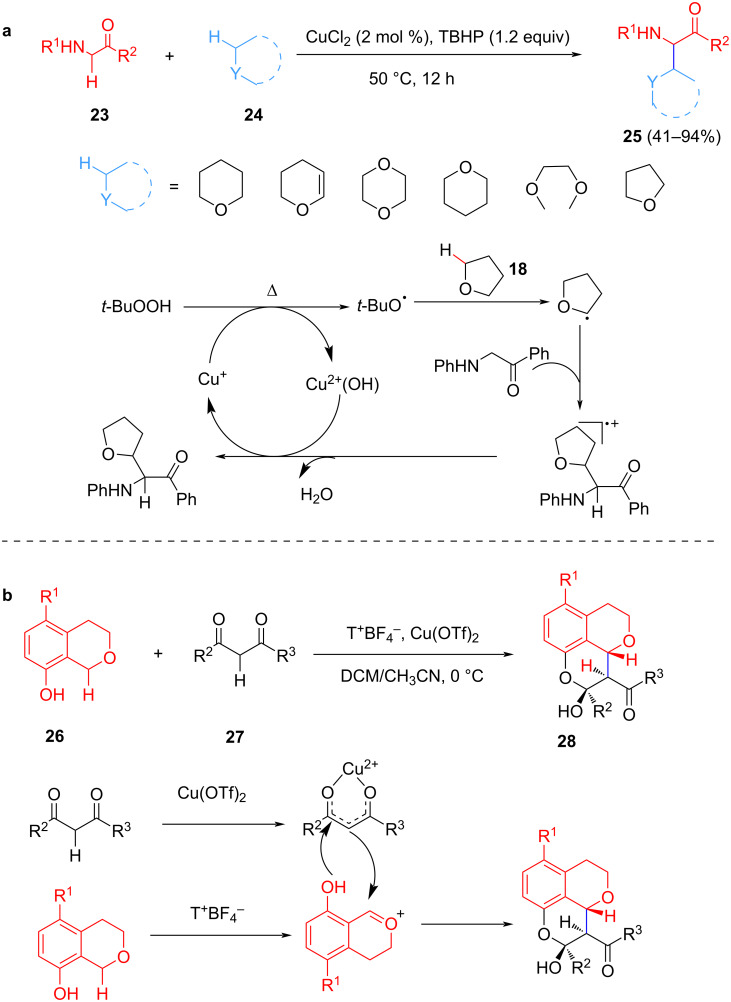
Cu-catalyzed CDC of C(sp^3^)–H/C(sp^3^)–H bonds.

In 2014, Li et al. reported a CuCl_2_-catalyzed cross-dehydrogenation coupling reaction of C(sp^3^)–H bonds adjacent to an ether oxygen and the C(sp^3^)–H bonds at the α-position of a carbonyl functionality in the presence of TBHP as oxidant (Scheme 7a) [56]. Various α-ether-alkylated aminocarbonyl compounds were synthesized via this method and it could be extended to various α-amino ketones, α-amino esters, and α-amino amides. The mechanism of these coupling reactions is very similar and is initiated by the attack of the radical initiator to the ether to obtain the corresponding ether radical species. The coupling product is accessed through a single electron transfer (SET) and other transformations.

In 2019, Tu et al. established a highly efficient Cu-catalyzed cross-dehydrogenative coupling to access a tricyclic chromane nucleus from 8-hydroxyisochromanes and 1,3-dicarbonyl compounds in the presence of Cu(OTf)_2_ and T^+^BF_4_^−^ (Scheme 7b) [57]. The strategy has a wide range of applications and is highly diastereoselective, making it an attractive strategy for synthesizing related natural products. The role of copper is to activate the 1,3-dicarbonyl compounds through complexation that leads to a highly diastereoselective nucleophilic addition.

**Scheme 8:**
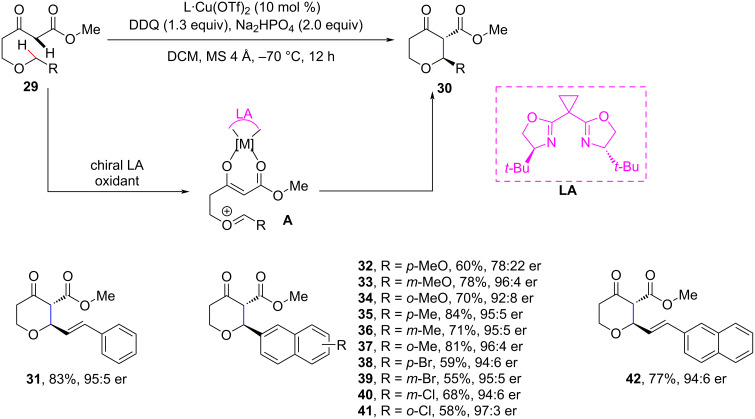
Cu-catalyzed synthesis of chiral 2-substituted tetrahydropyrans.

Scheidt et al. reported an enantioselective Cu-catalyzed intramolecular cross-dehydrogenative coupling approach to substituted tetrahydropyrans with excellent yields and stereoselectivity (Scheme 8) [58]. The mechanism of this reaction differs from the previously reported ones and proceeds through the in situ generation of nucleophilic and electrophilic partners which provides new opportunities for enantioselective oxocarbenium ion-driven CDC processes.

**Scheme 9:**
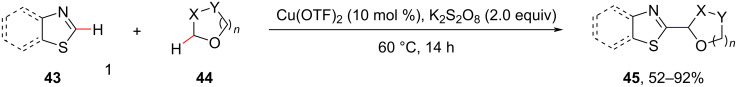
CDC of thiazole with cyclic ethers.

Due to an adjacent C=C bond, various conjugated alkenyl C–H bonds can also be activated to construct functionalized ethers. In 2013, Wang et al. achieved a mild Cu(OTf)_2_-catalyzed CDC of (benzo)thiazoles with cyclic ethers in the presence of K_2_S_2_O_8_ (Scheme 9) [59]. The catalytic system is also suitable for benzothiazole, in which benzothiazole compounds have higher reactivity and regioselectivity than thiazole.

**Scheme 10:**
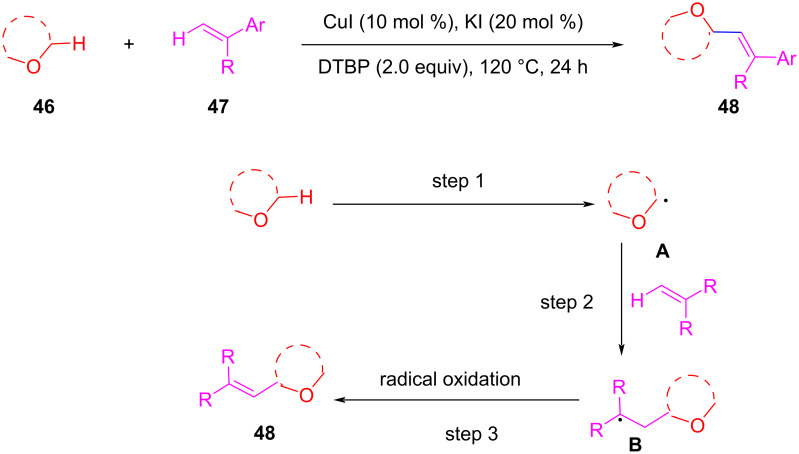
Cu(I)-catalyzed oxidative alkenylation of simple ethers.

In 2014, Lei et al. successfully realized the copper-catalyzed oxidative alkenylation of simple ethers to construct allyl ethers in the presence of di-*tert*-butyl peroxide and KI (Scheme 10) [60]. The oxidative olefination of simple ethers might undergo the following three successive steps: (1) the formation of an α-carbon-centered radical **A** from simple ethers, (2) addition of the α-carbon-centered radical to olefins generating radical **B**. This step is one of the classical transformations of radicals and has been proved in many reports, and (3) oxidation of radical **B** to provide the corresponding alkenyl products **48**.

**Scheme 11:**
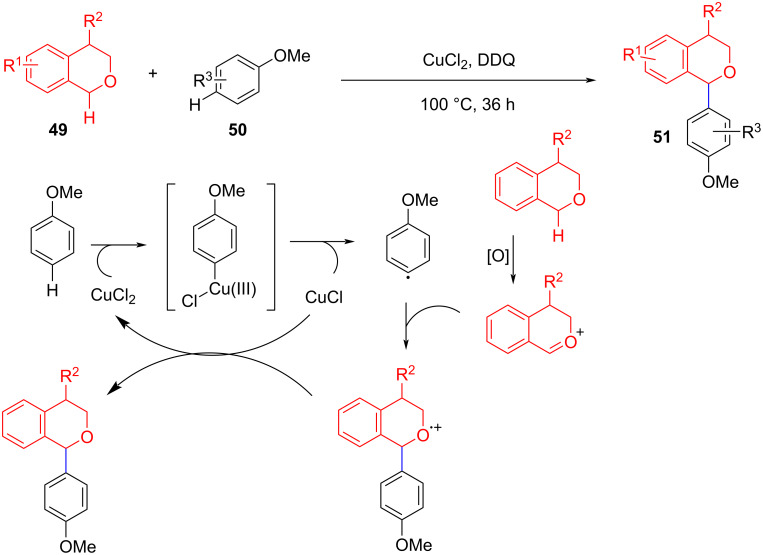
Cross-dehydrogenation coupling of isochroman C(sp^3^)–H bonds with anisole C(sp^2^)–H bonds.

In recent years, the CDC reaction of alkyl C(sp^3^)–H substrates with the C(sp^2^)–H of an aromatic, which allows the construction of highly diverse compounds, has attracted considerable attention. Todd et al. reported a method for the cross-dehydrogenation coupling of isochroman C(sp^3^)–H bonds with anisole C(sp^2^)–H bonds using CuCl as a catalyst and DDQ as an oxidant (Scheme 11) [61]. However, this method is not ideal for tolerating substrates with electron-donating substituents (such as 1-methylisochroman, 3-methoxyanisole). Mechanism experiments showed that the coupling of aromatic ring radicals with ether oxygen ions produced an intermediate radical cation, which achieves a catalytic cycle through the Cu center.

**Scheme 12:**
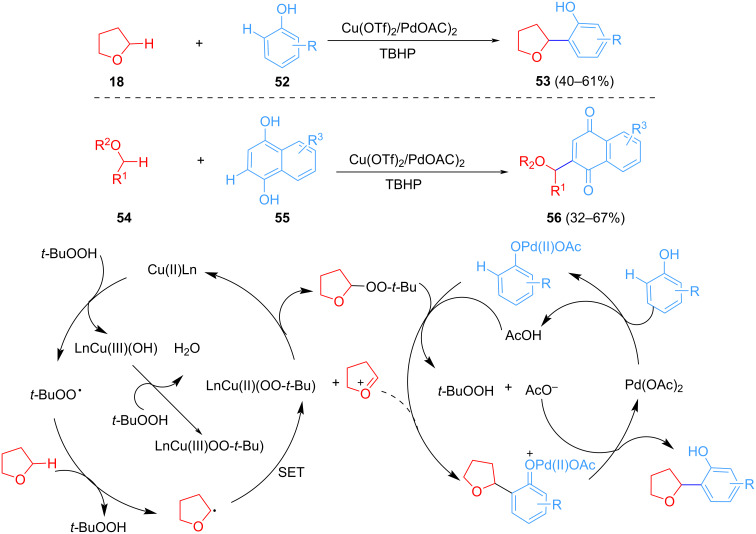
Pd(OAc)_2_/Cu(OTf)_2_-catalyzed arylation of α-C(sp^3^)–H bonds of ethers.

Lee et al. disclosed TBHP as an oxidant and Pd(OAc)_2_/Cu(OTf)_2_ as the catalyst to achieve the CDC of THF and phenol C(sp^2^)–H (Scheme 12) [62]. The role of Pd may be through the formation of a Pd(II) phenolic acid salt from phenol and Pd(OAc)_2_ to improve the reactivity of phenol. Subsequently, a more complex C(sp^2^)–H component was employed as a coupling substrate to functionalize the ether α-C–H bond.

**Scheme 13:**
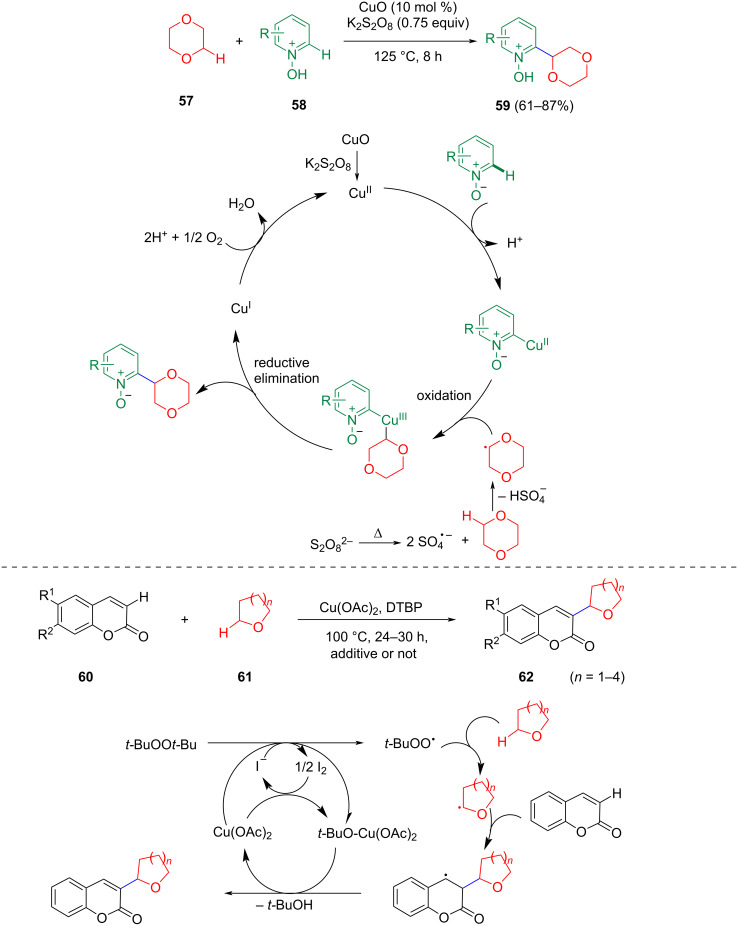
Cu-catalyzed C(sp^3^)–H/C(sp^2^)–H activation strategies to construct C(sp^3^)–C(sp^2^) bonds.

In the presence of Cu(II), the C(sp^2^)–C(sp^3^) coupling of pyridine *N*-oxides and coumarins with cyclic ethers could be achieved under mild conditions (Scheme 13) [63–64]. These reactions do not all follow the reaction mechanism of the oxidative olefination of simple ethers. The role of copper is mainly to activate the C(sp^2^)–H bond by coordination or activating the oxidant to achieve the reaction cycle.

**Scheme 14:**
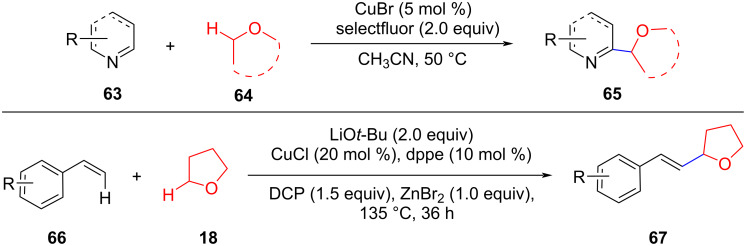
Cu(I)-catalyzed C(sp^2^)–H alkylation.

Subsequently, Li and Ahmad et al. reported a Cu(I)-catalyzed C(sp^2^)–H activation and ether formation of C(sp^2^)–C(sp^3^) bonds via CDC, respectively (Scheme 14) [65–66]. The isotope, radical detection, and other control experiments confirmed that the reaction proceeded through a radical oxidation process. The reaction of most substituted olefins with cyclic ethers afforded the corresponding target products with excellent yields, which provides a simple, novel, and efficient pathway to afford allyl ethers.

**Scheme 15:**
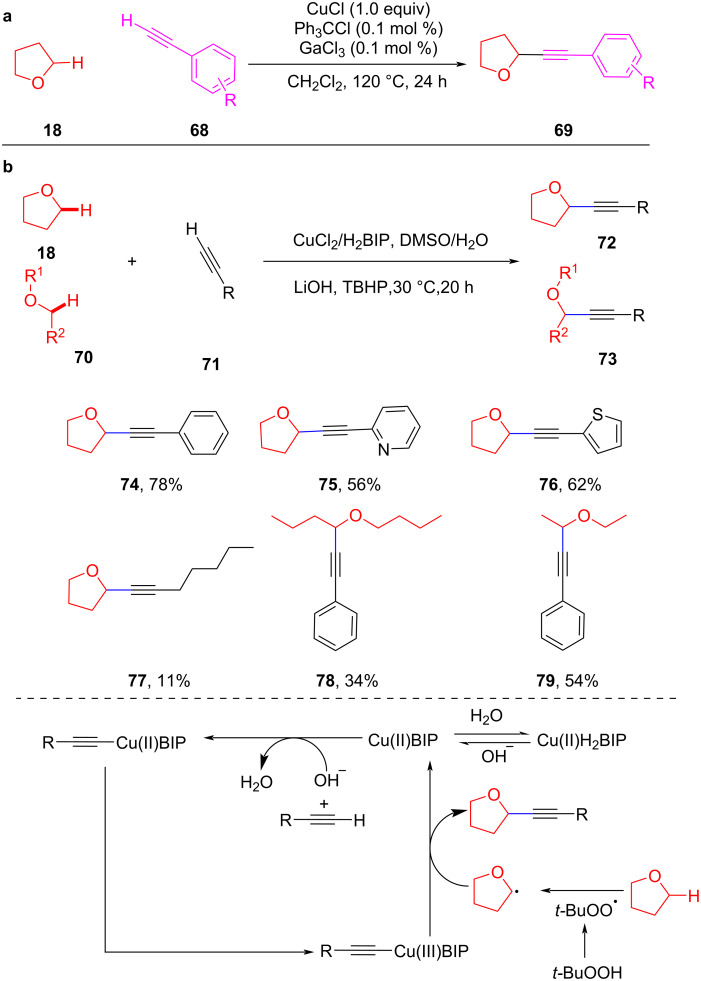
Cu-catalyzed C(sp^3^)–H/C(sp)–H activation to construct C(sp^3^)–C(sp) bonds (H_2_BIP: 2,6-bis(benzimidazol-2′-yl)pyridine).

Alkyne C(sp)–H bonds are reactive, and the challenge in the cross-coupling of C(sp)–H and C(sp^3^)–H bonds is to control chemoselectivity. In this context, Liu et al. reported a Cu(I)**/**Ga(III)-catalyzed trityl ion-mediated direct CDC of the C(sp^3^)–H bond of THF with C(sp)–H bonds of terminal alkynes at room temperature (Scheme 15a) [67]. The ability to tune the reactivity of the trityl ion rationally improves the approach with excellent regio- and diastereoselectivity for the unsymmetric ethers. In 2018, Ye et al. reported a CDC reaction to form C(sp)–C(sp^3^) coupling products from terminal alkynyl aldehydes with ethers in the presence of CuCl_2_ and TBHP (Scheme 15b) [68]. The reaction is compatible with various functional groups including cyclic ethers and open chain ethers. Studies on the reaction mechanism showed that the reaction is a catalytic cycle involving a radical process, and the cleavage of the C(sp^3^)–H bond in the ether substrates which produces α-alkyl radicals is the rate-determining step.

#### Fe-catalyzed reactions

Iron is a transition metal with abundant reserves, low price, and non-toxicity, which shows many characteristics in catalytic processes, such as the properties of transition metals and Lewis acids [69–72]. These advantages make iron salts attractive catalysts or reagents in chemical transformations and are considered ideal materials for developing catalysts [73].

**Scheme 16:**
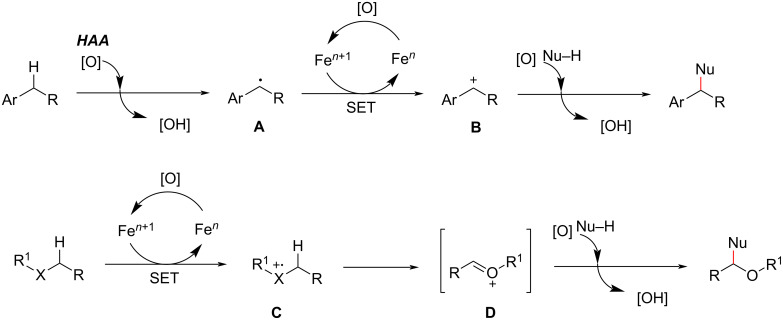
Fe-catalyzed CDC reaction pathways.

Fe-catalyzed CDC reactions have achieved remarkable achievements in recent years [74–77], which can directly activate inert C–H bonds to construct C–C bonds. Fe-catalyzed CDC reactions mainly follow the mechanism shown in Scheme 16. An oxidant abstracts a hydrogen from the C–H bond to generate a carbon-centered radical **A**. Then, through a single-electron transfer (SET) process, the carbocation intermediate **B** is generated, which is attacked by a nucleophile to afford the target product. Further, C–H bonds in the *ortho*-position of a heteroatom are activated through a SET pathway generating a radical cation **C**, which is easily deprotonated by an oxidant to generate a carbocation **D**. Finally, the nucleophile attacks the carbocation **D**, to obtain the final coupled product. The deprotonation of the nucleophile occurs before or after the attack on the carbocation intermediate, depending on the acidity of the nucleophile.

**Scheme 17:**
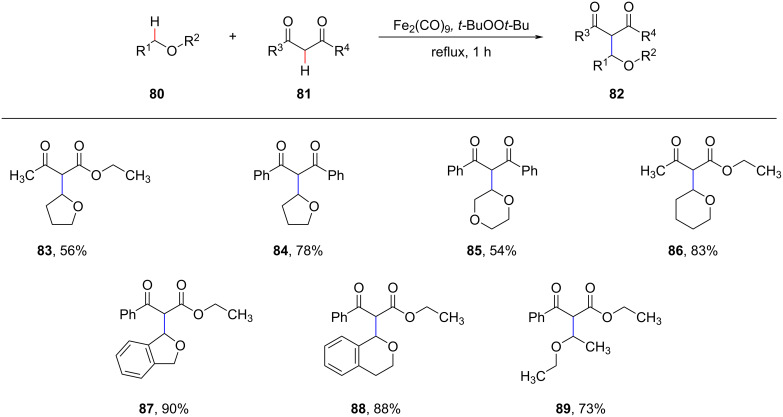
Fe_2(_CO)_9_-catalyzed functionalization of C–H bonds.

In 2008, Li et al. reported that Fe_2_(CO)_9_ as a catalyst in combination with di-*tert*-butyl peroxide (DTBP) as an oxidant enables the CDC of the C(sp^3^)–H bond in the α-position to oxygen of various ethers with the active methylene C(sp^3^)–H bond in 1,3-diketones (Scheme 17) [78]. This method can generate various functionalized molecules and is expected to have broad applications in synthesis.

**Scheme 18:**
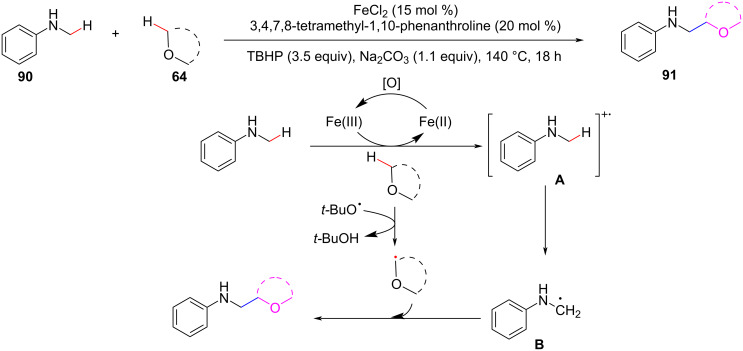
Ligand-promoted Fe-catalyzed CDC reaction of *N*-methylaniline with ethers.

In 2019, Cai et al. developed a regioselective ligand-promoted CDC reaction between unactivated C(sp^3^)–H/C(sp^3^)–H bonds (Scheme 18) [79]. Different types of C(sp^3^)–H bond substrates, including cycloalkanes, cyclic ethers, and toluene derivatives without any directing groups could be used as coupling partners. The ligand acts as an activator of the catalyst to promote the reaction, and the iron-bound anion plays a crucial role in catalysis. This reaction might occur via a radical pathway, with the iron catalyst playing a significant role in electron transfer, and TBHP acting as both an oxidant and a radical initiator.

**Scheme 19:**
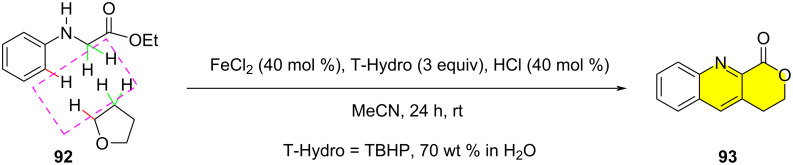
Fe-catalyzed CDC of C(sp^3^)–H/C(sp^3^)–H bonds.

In 2015, Wang et al. reported the synthesis of quinoline lactones by the double oxidative dehydrogenation (DOD) reaction between glycine derivatives and tetrahydrofuran using the FeCl_2_/HCl/TBHP system (Scheme 19) [80]. This practical coupling method allows the efficient alkylation of aromatic rings, can directly afford pharmaceutically significant heterocycles, and the raw materials and iron catalysts are safe and readily available.

**Scheme 20:**
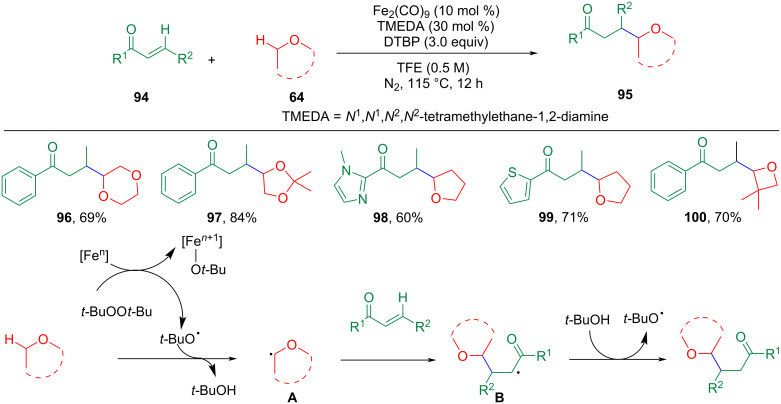
Fe-catalyzed hydroalkylation of α,β-unsaturated ketones with ethers.

In 2017, Xu, Loh, and co-workers, demonstrated an iron-catalyzed hydroalkylation reaction of α,β-unsaturated ketones **94** with ethers [81]. Contrary to what was obtained for the alkylation of coumarin at the carbonyl α-position, vinyl ketone undergoes Michael addition and ether addition at the β-position of the carbonyl (Scheme 20). The reaction delivered various alkylation products in good to excellent yields with Fe_2_(CO)_9_ as a catalyst and *N*^1^,*N*^1^,*N*^2^,*N*^2^-tetramethylethane-1,2-diamine (TMEDA) as bidentate ligand. A gram-scale alkylation reaction showed that the new procedure has excellent potential for synthetic applications. The mechanism study shows that radicals are the starting point of the coupling reaction. First, ether radicals **A** are produced by the reaction with a *tert*-butoxyl radical. The ether radicals **A** could undergo a conjugate addition to the 1,4-unsaturated system to form a new radical intermediate **B**. This intermediate extracts a hydrogen from *tert*-butanol to form a neutral target product and regenerates the *tert*-butoxyl radical to complete the entire catalytic cycle. The formation of hydrogen bonds between the oxygen of the carbonyl group and the hydrogen of the 2,2,2-trifluoroethanol (TFE) reduces the activation energy of the radical reaction and improves the coupling efficiency.

**Scheme 21:**
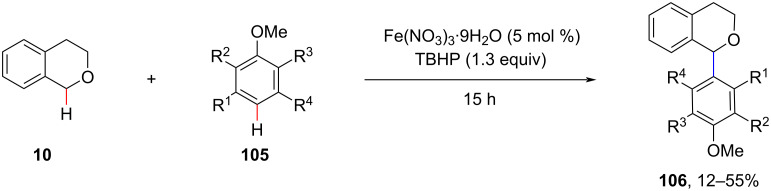
Solvent-free Fe(NO_3_)_3_-catalyzed CDC of C(sp^3^)–H/C(sp^2^)–H bonds.

In 2010, Schnürch et al. utilized Fe(NO_3_)_3_ as the catalyst and TBHP (*tert*-butyl hydroperoxide) as the oxidant to realize the CDC of C(sp^3^)–H/C(sp^2^)–H bonds under solvent-free conditions (Scheme 21) [82]. However, the desired coupling products were obtained in low to moderate yields.

**Scheme 22:**
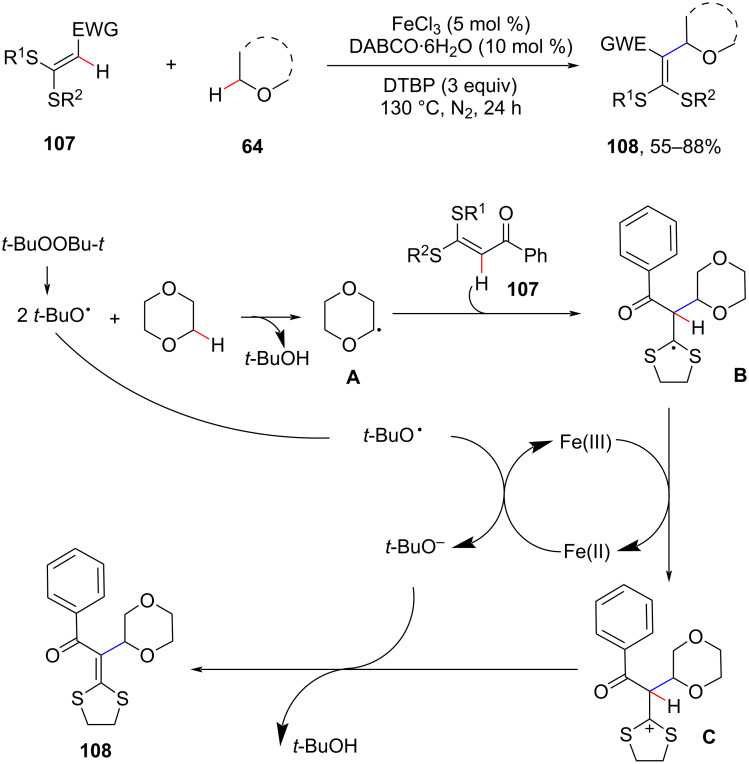
Alkylation of disulfide compounds to afford tetrasubstituted alkenes.

The C–H oxidative alkylation of *S*,*S*-functionalized internal olefins was achieved by a C(sp^2^)–H/C(sp^3^)–H cross-coupling reaction using DTBP as oxidant and DABCO·6H_2_O as an additive in the presence of FeCl_3_ (Scheme 22) [83]. The reaction provides a convenient route to tetrasubstituted alkenes and proceeds via a typical radical coupling process. Initially, ether **64** interacts with *tert*-butoxyl radicals via hydrogen atom transfer reaction to generate radical **A** with release of *tert*-butyl alcohol. Subsequently, the radical **A** adds to the C=C bond of α-oxo ketene dithioacetal **107** to form radical **B**, which further reacts with Fe(III) to form cationic intermediate **C** and Fe(II) through a single electron transfer (SET) process. Subsequent abstraction of a proton from species **C** by a basic *tert*-butoxy anion generated from DTBP affords the product.

**Scheme 23:**
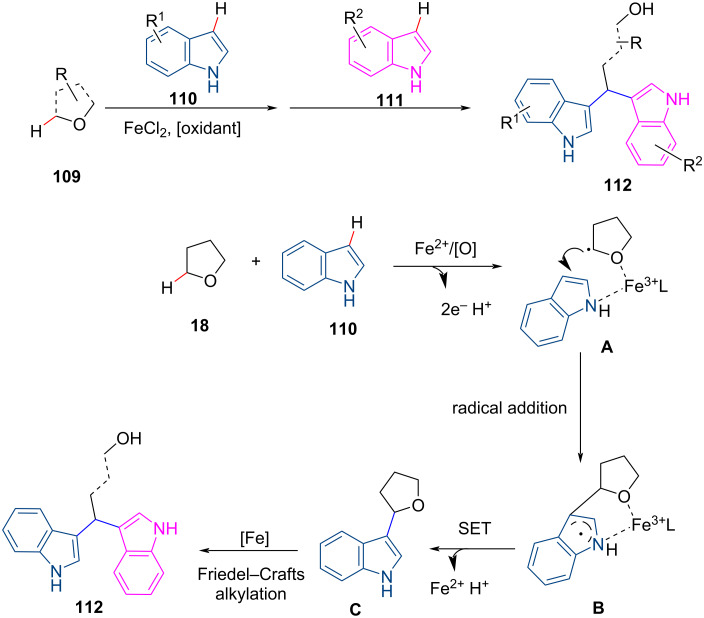
Fe-catalyzed formation of 1,1-bis-indolylmethane derivatives.

Li et al. reported Fe-catalyzed CDC reactions of C(sp^2^)–H bonds of indoles and C(sp^3^)–H bonds of ethers to obtain symmetric and asymmetric 1,1-bis-indolylmethane derivatives (Scheme 23) [84]. The reaction proceeds through the tandem oxidative coupling of the C–O bond and cleavage of the C–H bond. Fe plays a dual role in catalysing the C–C bond coupling and C–O bond cleavage as Lewis acid catalyst. The authors demonstrated that the introduction of the two indoles occurs in two distinct steps, a radical process and a Friedel–Crafts alkylation reaction.

**Scheme 24:**
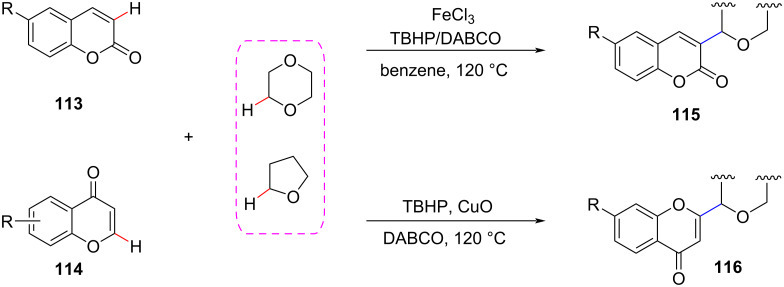
Alkylation of coumarins and flavonoids.

Coumarin and flavonoid derivatives are very valuable precursors in drug synthesis. In 2015, Ge et al. developed the regioselective and atom-economical CDC reaction of coumarin and flavonoids with different ethers through a C(sp^3^)–H activation process and obtained two novel ether-substituted derivatives (Scheme 24) [85]. This method can introduce ether substituents at the electron-rich α-position of coumarin and the β-H position of flavonoids.

**Scheme 25:**
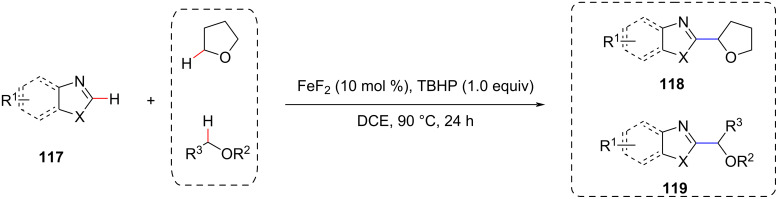
Direct CDC α-arylation of azoles with ethers.

In the same year, Correa et al. reported the FeF_2_-catalyzed direct α-arylation of azoles with ethers (Scheme 25) [86]. This approach was suitable for assembling a wide variety of functionalized heterocycles, representing an attractive strategy for the C–H alkylation of azoles. The authors also discussed the reaction mechanism supported by DFT calculations and concluded that FeF_2_ plays an important redox role in assisting the cleavage of oxidants and the oxidation of carbon radicals to cationic intermediates of oxygen.

**Scheme 26:**
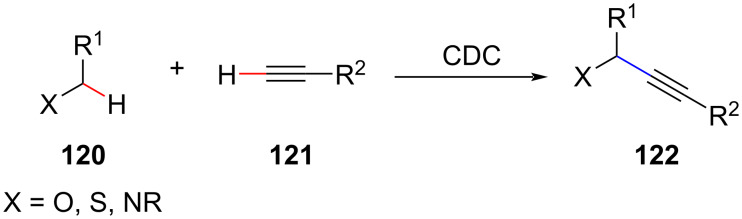
CDC of terminal alkynes with C(sp^3^)–H bonds adjacent to oxygen, sulfur or nitrogen atoms.

CDC reactions between C(sp^3^)–H/C(sp)–H bonds catalyzed by iron have been reported to afford internal alkynes from substrates with the C(sp^3^)–H bond mainly located in the α-position to N, O, or S atoms. This method provides a direct and atom-economical alternative for the construction of structurally complex alkyne compounds (Scheme 26) [87]. In addition to iron, various other transition metals such as Cu, Pd, and Ag are also suitable to catalyze the reaction.

**Scheme 27:**
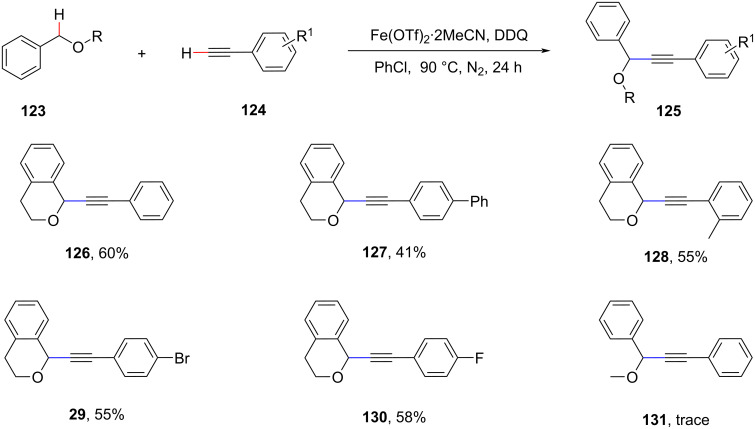
Alkylation of terminal alkynes.

In 2012, Xiang et al. reported the CDC of aryl ethers with C(sp^3^)–H bonds adjacent to the ether oxygen with terminal alkyne C(sp)–H bonds, which provides a new approach for the construction of the C(sp^3^)–C(sp) bonds (Scheme 27) [88]. This route provides an environmentally friendly and practical approach to alkyl-substituted alkynes.

#### Co-catalyzed reactions

In recent years, cobalt has exhibited great application potential as a cross-dehydrogenation coupling catalyst due to its low price, environmentally friendliness, and unique catalytic behavior [89]. However, there are only a few examples of cobalt catalysis in CDC reactions.

**Scheme 28:**
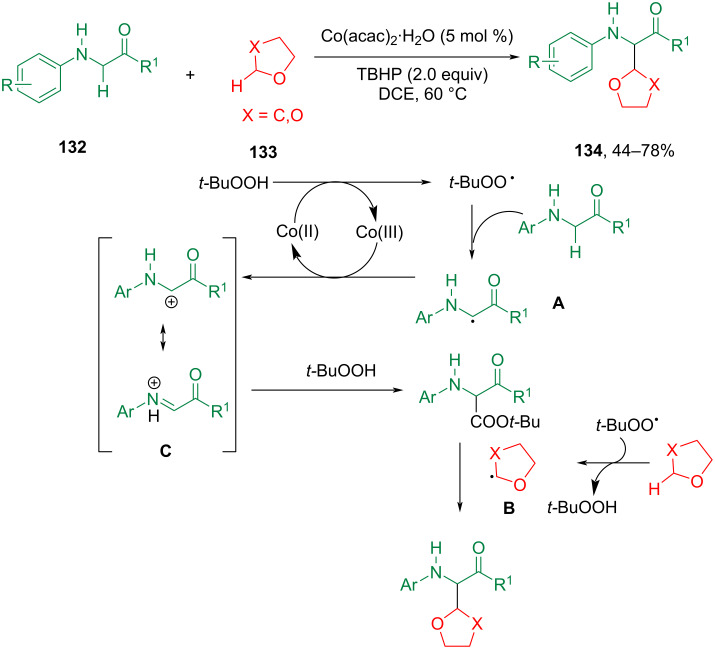
Co-catalyzed functionalization of glycine esters.

Limited by the activity of Co catalysts, there are few examples of Co-catalyzed reactions involving ether C(sp^3^)–H bond activation. The Co-catalyzed C(sp^3^)–C(sp^3^) CDC of glycine and peptide derivatives with THF was developed by Correa et al. (Scheme 28) [90]. This study presents a cost-effective cobalt-catalyzed C(sp^3^)–H functionalization strategy for α-aminocarbonyl compounds. The method allows for the direct introduction of ethers into a diverse range of glycine derivatives. Importantly, the reaction conditions are base-free and mild (60 °C), allowing the preservation of chiral centers. The developed approach enables the synthesis of various α-functionalized glycine derivatives, which play a crucial role in proteomics. The work offers a novel perspective on cobalt-catalyzed C–H functionalizations.

**Scheme 29:**
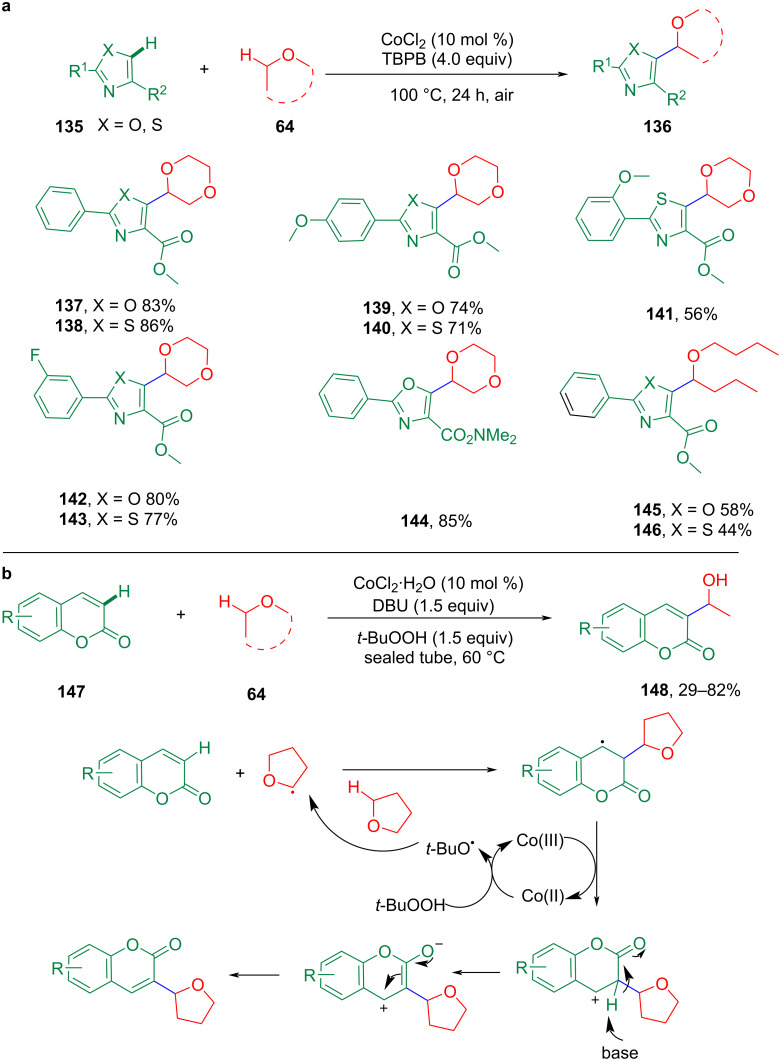
Co-catalyzed construction of C(sp^2^)–C(sp^3^) bonds.

As the C(sp^2^)–C(sp^3^) bond is the most common building unit in organic skeletons, the Co-catalyzed formation of C(sp^2^)–C(sp^3^) bonds via C–H bond functionalization, notably via the CDC strategy, is an interesting and challenging research field that has attracted much attention in recent years. In 2017, the Co-catalyzed CDC for the C5-alkylation of oxazole/thiazole substrates with ethers afforded functionalized ethers in moderate to good yields (Scheme 29a) [91]. In 2016, Du et al. demonstrated that the construction of C(sp^2^)–C(sp^3^) bonds also proceeded smoothly with coumarins and cyclic or open-chain alkyl ethers in the presence of DBU under relatively mild conditions (Scheme 29b) [92].

**Scheme 30:**
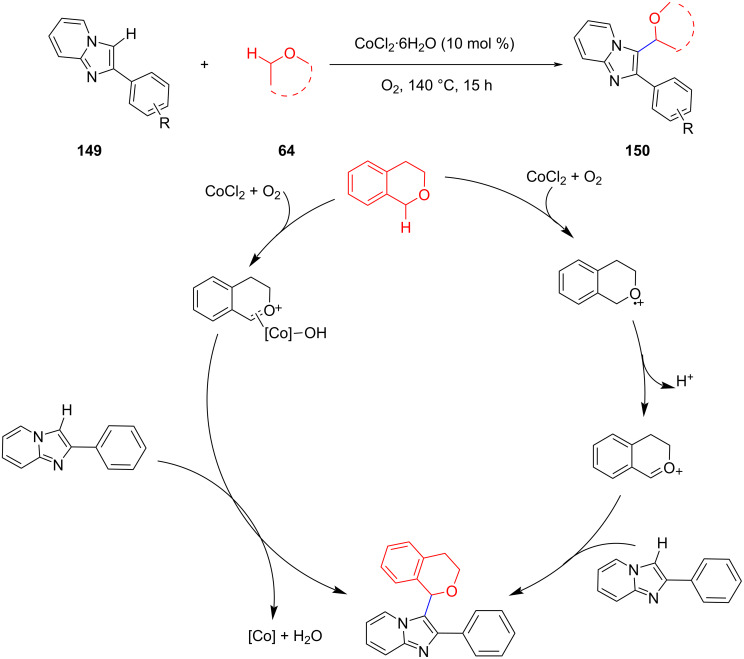
Co-catalyzed CDC of imidazo[1,2-*a*]pyridines with isochroman.

In 2018, Wang et al. developed the cobalt-catalyzed oxidative CDC reaction of 2-arylimidazo[1,2-*a*]pyridines with isochroman using molecular oxygen as an oxidant (Scheme 30) [93]. These reactions involved a metal-triggered oxidation of the ether substrate to obtain the corresponding radical or oxonium ion as the key intermediate to obtain the final coupling product.

**Scheme 31:**
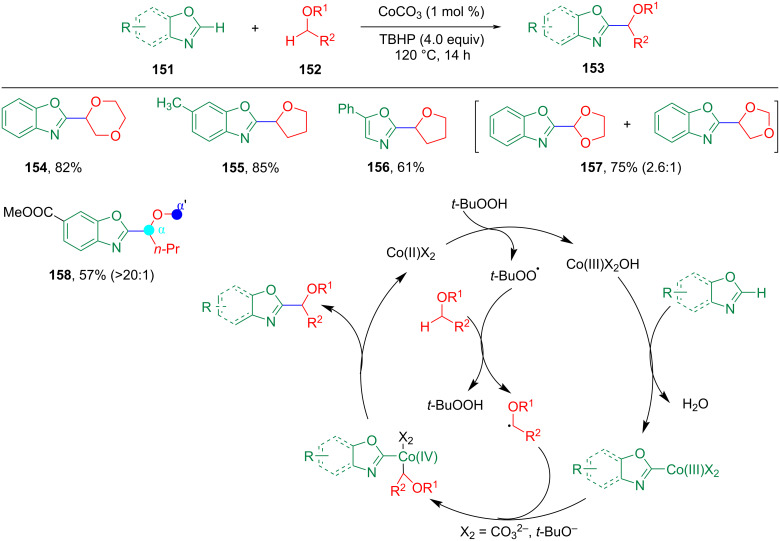
Co-catalyzed C–H alkylation of (benz)oxazoles with ethers.

Subsequently, some novel Co-catalyzed coupling mechanisms have been proposed. In 2016, Lu et al. reported that the Co/TBHP catalyst oxidation system achieved the alkylation of various azole compounds through the CDC reaction, which broadened the application range of azole substrates (Scheme 31) [94]. The reaction system is simple and the products can be obtained with medium or good yield. The directing functional groups in the substrate can be coordinated with metal catalysts to control the selectivity and improve the reactivity in metal-catalyzed or -mediated reactions. Therefore, controlling the regioselectivity of CDC reactions by directing groups is of great interest [95–96].

**Scheme 32:**
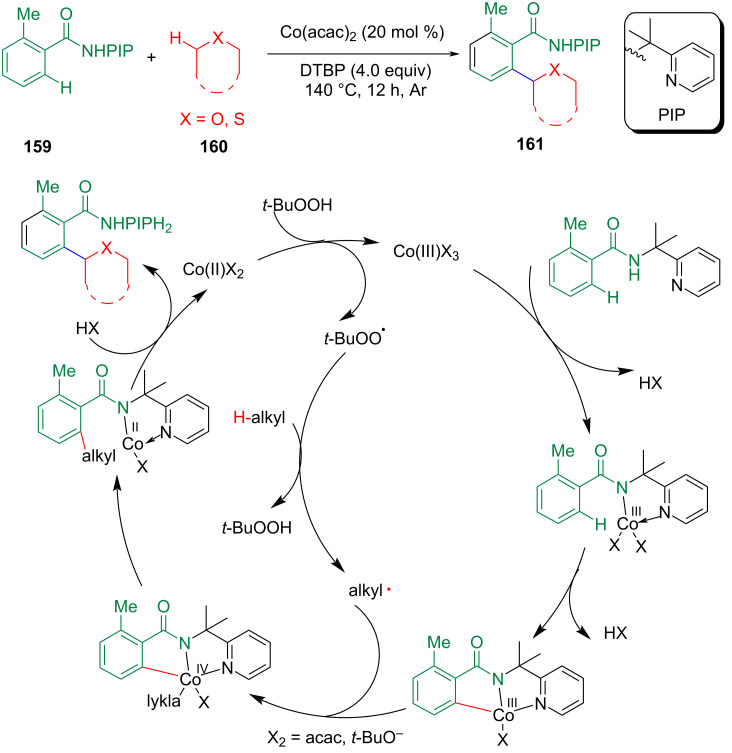
Cobalt-catalyzed CDC between unactivated C(sp^2^)–H and C(sp^3^)–H bonds.

Li et al. reported a cobalt-catalyzed CDC between unactivated C(sp^2^)–H and C(sp^3^)–H bonds by the *ortho*-alkylation reaction of aromatic carboxamides containing (pyridin-2-yl)isopropylamine (PIP–NH_2_) as an *N*,*N*-bidentate directing group (Scheme 32) [97]. The mechanism study showed that these reactions were a Co(III/IV/II) catalytic cycle, and the coordination of Co with the substrate achieved the activation of the C(sp^2^)–H bond.

#### Other non-noble metal-catalyzed reactions

**Scheme 33:**
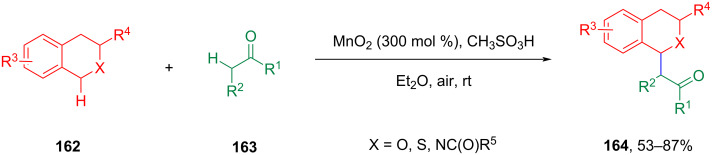
MnO_2_-catalyzed CDC of the inactive C(sp^3^)-H.

In 2013, Liu et al. reported that MnO_2_ could catalyze the CDC of the benzylic C(sp^3^)–H bond in benzyl ethers with α-carbonyl C(sp^3^)–H bonds in the presence of air at room temperature (Scheme 33) [98]. This pathway involves the presence of methanesulfonic acid and a large amount of metal oxide to obtain the target product with moderate to good yield.

**Scheme 34:**
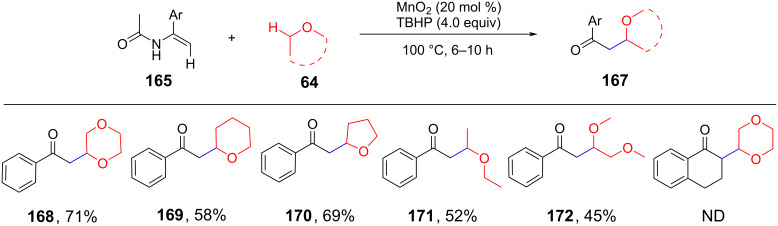
Oxidative cross-coupling of ethers with enamides.

In 2015, a MnO_2_-catalyzed sequential oxidative alkylation of C(sp^3^)–H/C(sp^2^)–H CDC and hydrolysis of enamides with ethers was reported by Xu et al. (Scheme 34) [99]. The CDC reaction was initiated by a radical oxidative addition of C(sp^2^)–H and the final product was obtained by amide hydrolysis. The strategy provides a simple and practical method for synthesizing β-oxoketones.

**Scheme 35:**
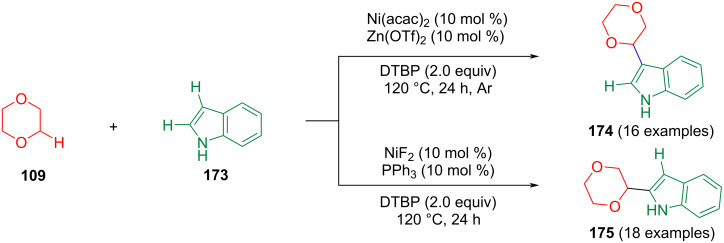
Ni(II)-catalyzed CDC of indoles with 1,4-dioxane.

In 2015, Cai et al. reported a novel CDC of C(sp^3^)–H and C(sp^2^)–H bonds in indoles with 1,4-dioxane C(sp^3^)–H bonds via Ni(II) catalysis (Scheme 35) [100]. The selectivity of the reaction is determined by the catalyst used, which provides an efficient strategy for the selective construction of cyclic ethers containing heteroaromatic structures.

**Scheme 36:**
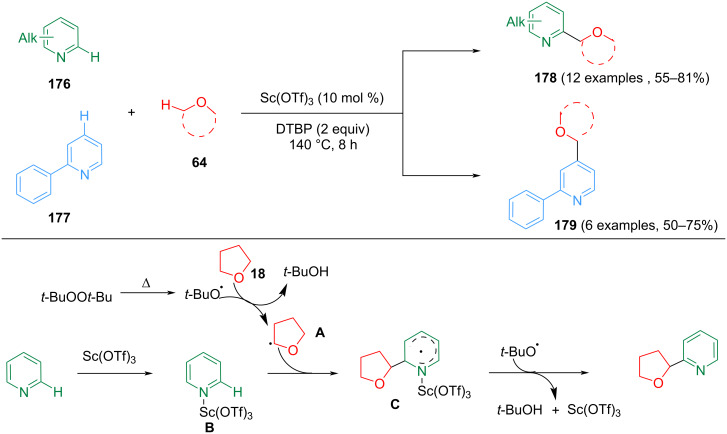
Chemo- and regioselective o*rtho-* or *para-*alkylation of pyridines.

Huang et al. reported a highly chemoselective and regioselective CDC between pyridines and ethers, which used Sc(OTf)_3_ as the catalyst and DTBP as the oxidant (Scheme 36) [101]. This strategy allowed the synthesis of a series of α-substituted pyridine derivatives. The control experiments showed that the mechanism may proceed via a radical pathway. Initially, a *tert*-butoxyl radical is generated by thermal decomposition. Then, the *tert*-butoxyl radical extracts an α-hydrogen atom from tetrahydrofuran to form tetrahydrofuran radical **A**. Sc(OTf)_3_ as a Lewis acid activates pyridine forming the pyridine complex **B**. Then, radical **A** adds to the more electron-deficient position of the pyridine ring in complex **B** to obtain pyridine radical **C**, which aromatizes through *tert*-butoxyl radical-mediated extraction of hydrogen to afford the desired 2-substituted pyridine and regenerate Sc(OTf)_3_.

**Scheme 37:**
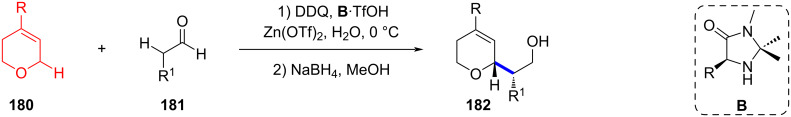
Asymmetric CDC of 3,6-dihydro-2*H*-pyrans with aldehydes.

In 2019, Liu et al. first reported an enantioselective CDC of dihydropyrans (DHPs) and aldehydes in the presence of Zn(II) (Scheme 37) [102]. The method has good enantioselectivity and functional group tolerance and provides a practical and economical route towards a series of enantiopure α-substituted DHPs through CDC, through an in situ NaBH_4_ reduction two-step sequence.

**Scheme 38:**
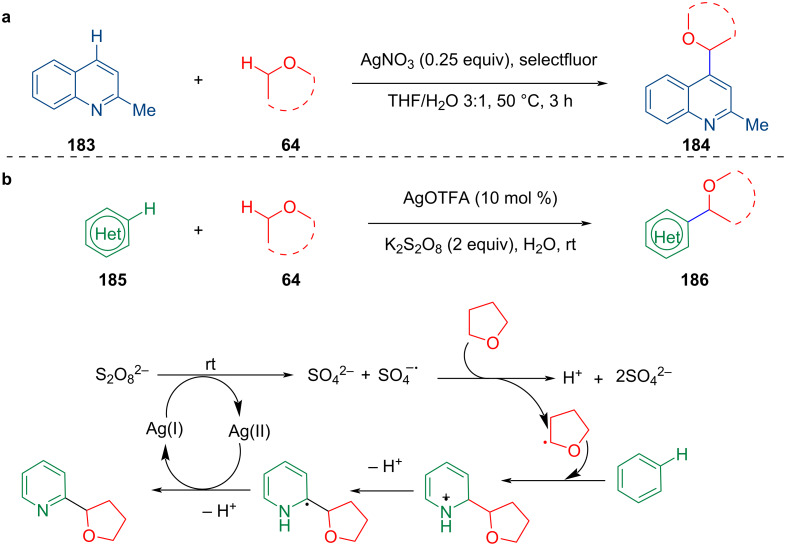
CDC of heterocyclic aromatics with ethers.

In recent years, a Ag-catalyzed cross-dehydrogenative coupling of aromatic C(sp^2^)–H bonds with ethers has also been developed. In 2018, Wang et al. reported that the AgNO_3_-promoted CDC of quinaldine (**183**) with ethers afforded alkylated quinoline derivatives in the presence of selectfluor as a mild oxidant (Scheme 38a) [103]. Subsequently, Li et al. developed the CDC of heterocyclic aromatics with simple ethers mediated by AgOTFA to construct C(sp^2^)–C(sp^3^) bonds. The reaction proceeds under mild conditions (room temperature) with a wide range of substrates (Scheme 38b) [104]. The reaction mechanism is similar to the CDC reaction of simple ethers by transition-metal catalysis. First, Ag triggers the oxidant to produce oxidant radicals, and the corresponding ether radicals are obtained by extraction of H atoms from the ether substrates by the oxidant radicals. Then, the addition of the radicals and elimination occurs to give the target coupling product.

**Scheme 39:**
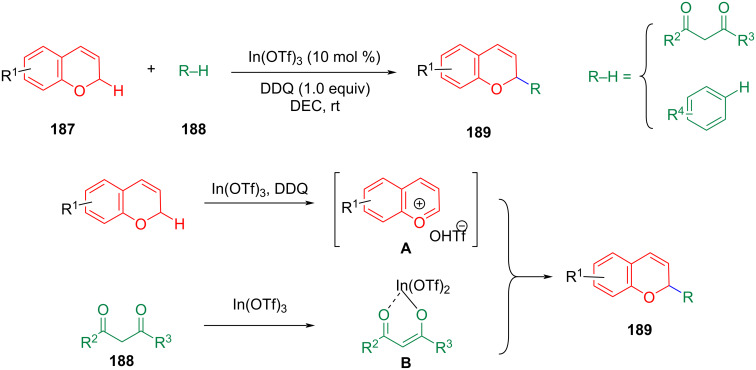
Indium-catalyzed alkylation of DHPs with 1,3-dicarbonyl compounds.

Liu et al. reported a novel In-catalyzed coupling of benzopyrans with 1,3-dicarbonyl moieties and aryl rings using dichloro-5,6-dicyano-1,4-benzoquinone (DDQ) as the oxidant (Scheme 39) [105]. Under the established standard conditions, various dicarbonyl compounds could be coupled with 2,4-disubstituted 3,6-dihydro-2*H*-pyrans (DHPs). The DHP motif is a structural component of several bioactive natural products and synthetic drugs with antioxidant, antipsychotic, antibacterial, antifungal, antiviral, and anticancer activities [106–107]. The current synthesis method depends on the reaction of organic borane with epoxy carbonyl compounds. Using the CDC reaction can save some synthesis steps [105]. Based on DDQ-mediated oxidative C–H functionalization of benzyl ethers, the mechanism can be described as a single electron transfer (SET) from the DHP substrates to DDQ, a hydrogen atom transfer (HAT), and counter anion exchange of In(OTf)_3_ might happen to generate ion pair **A**. In(OTf)_3_ coordinates with the carbonyl oxygen atoms in dimethyl malonate **188** to provide activated complex **B** for subsequent addition to **A** furnishing product **189**.

**Scheme 40:**
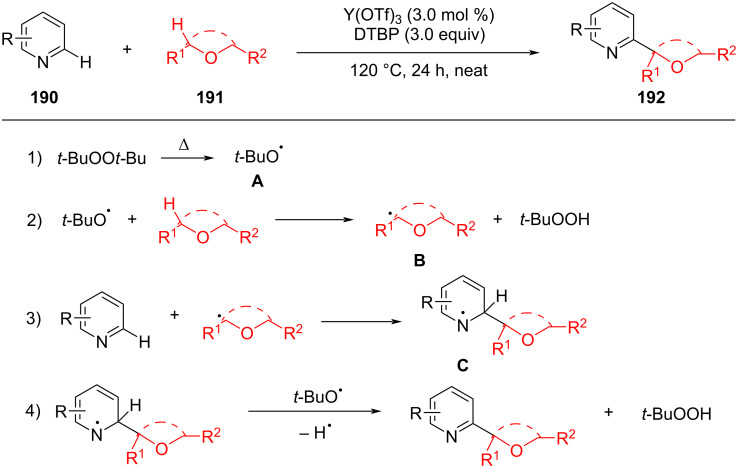
Rare earth-metal-catalyzed CDC reaction.

With the development of the CDC reaction, the catalytic effect of rare earth metals on CDC has also been explored. Xing et al. developed an Y(OTf)_3_-catalyzed CDC reaction of ethers, especially open chain ethers with pyridine derivatives (Scheme 40) [108]. The method has the advantages of simple operation, wide substrate range, and atom economy. It provides a new strategy for constructing functionalized pyridines. The reaction undergoes the following four processes: initially, DTBP is decomposed into two *tert*-butylperoxyl radicals **A** under heat. Then, the *tert*-butylperoxyl radical converts the ether into a carbon-centered radical **B** which then combines with 2-methylpyridine to obtain radical intermediate **C**. Oxidation of intermediate **C** by radical **A** then furnishes the product.

### Photocatalyzed CDC reactions

**Scheme 41:**
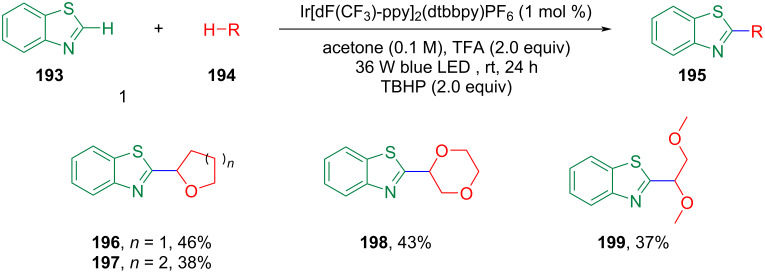
Visible-light-driven CDC of cycloalkanes with benzazoles.

In recent years, visible-light-driven photocatalytic processes have been considered influential in functionalizing unactivated C(sp^3^)–H substrates, including ethers [109–114]. In 2018, Wang et al. reported the photocatalytic CDC α-alkylation of N-heteroarenes in acetone solution, using noble-metal Ir as a photocatalyst to induce the reaction (Scheme 41) [115]. Subsequently, noble metals have been extensively studied as photocatalysts for CDC reactions [116–121] and these methods fill the gap of traditional thermocatalytic CDC reactions.

**Scheme 42:**
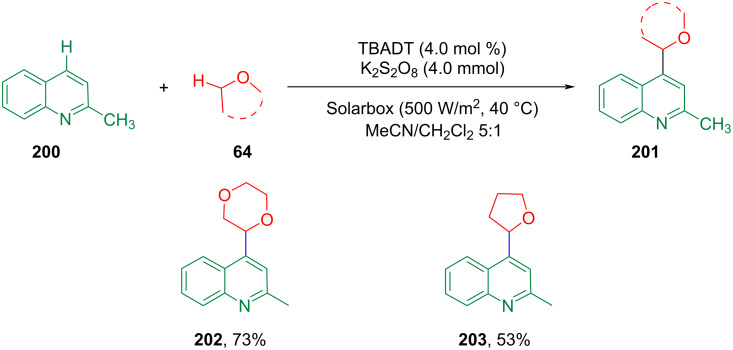
Photoinduced alkylation of quinoline with cyclic ethers.

In 2017, Ryu et al. developed tetrabutylammonium decatungstate (TBADT, (*n*-Bu_4_N)_4_[W_10_O_32_] as a photocatalyst to promote the sunlight-induced reaction and hydrogen transfer CDC of heteroaromatics and several H-donors (including ethers) under mild conditions (Scheme 42) [122]. There have been no previous reports using this catalyst for the alkylation of aromatics.

**Scheme 43:**
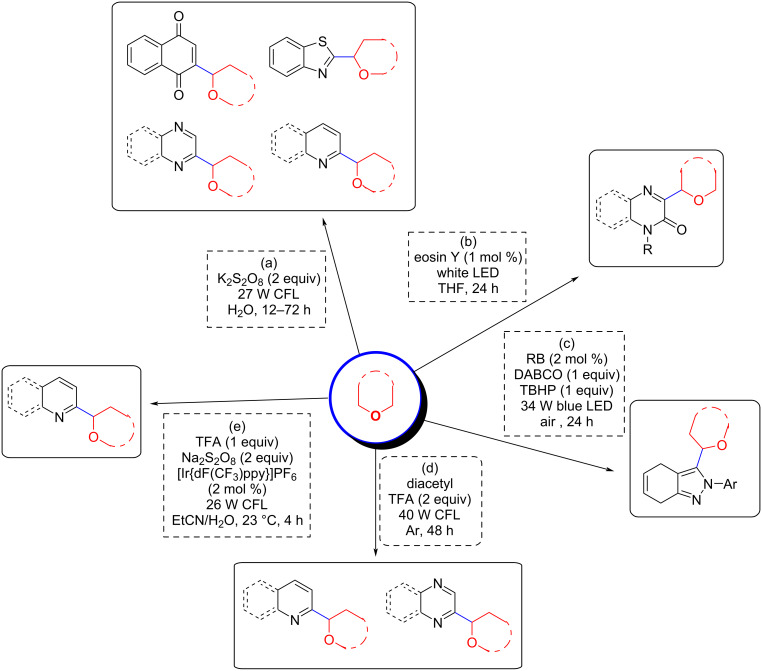
Photocatalyzed CDC reactions between α-C(sp^3^)–H bonds of ethers and C(sp^2^)–H bonds of aromatics.

Subsequently, various photocatalytic CDC methodologies involving ether α-C(sp^3^)–H and aromatic C(sp^2^)–H bonds were investigated and these are shown in Scheme 43. Shah et al. reported a catalyst-free CDC method using only 2 equivalents of K_2_S_2_O_8_ in H_2_O under irradiation with a 27 W CFL (Scheme 43a) [123]. In this reaction, both water and the light source played a key role, with lower yields or no product obtained when the reaction was performed without water or under other light source conditions such as 19 W CFL or irradiation with blue or green LEDs. This method is applicable to various heteroatom-containing compounds such as quinolines, pyrazines, pyridines, quinolines, isoquinolines, benzothiazoles, benzoquinones, etc. Under the reaction conditions, various ethers such as 1,4-dioxane, tetrahydropyran, tetrahydrofuran, diethyl ether, etc. are suitable substrates. Immediately thereafter, various photocatalytic catalysts were developed for this type of CDC reaction (Scheme 43b–e). Efficient CDC reactions could be achieved with 1 mol % of eosin Y in the absence of additional base or oxidizing agents. In this transformation, eosin Y may act as a direct hydrogen atom transfer photocatalyst (Scheme 43b) [124]. The CDC reaction between heterocyclic aromatics with α-C–H bonds of ethers was achieved under the irradiation of a 34 W blue LED using rose bengal (RB) as the organic photoredox catalyst, TBHP as oxidizing agent, and DABCO as the base (Scheme 43c) [125]. The wide scope of substrates, aerobic conditions, and gram-scale suitability are attractive features of this approach. Li et al. reported a new strategy for a metal-free CDC alkylation under mild conditions using 2,3-butanedione (diacetyl) as the hydrogen atom abstractor to extrude a hydrogen from the ether substrate to generate the radical intermediate which affords the products (Scheme 43d) [126]. Further, in the presence of [Ir{dF(CF_3_)ppy}_2_(dtbbpy)]PF_6_ as a photocatalyst, Na_2_S_2_O_8_ as oxidant, and TFA as an additive, under the irradiation of 26 W CFL at room temperature, the CDC reaction of various heterocyclic aromatics with α-C(sp^3^)–H bonds of ethers could be accomplished (Scheme 43e) [127]. At present, only a limited number of reports are available for electrocatalytic CDC reactions involving ether α-C(sp^3^)–H bonds and their susceptibility to electrocatalytic conditions, which hinders the application of electrocatalysis in this type of coupling reaction [128–130].

## Conclusion

In summary, Li et al. first proposed the concept of CDC, which now plays an essential role in organic synthesis. The atom- and step-economic CDC reaction can directly construct various C–C bonds from unreactive C–H substrates, including functionalized ethers. By reviewing these reactions, it can be seen that the reaction almost always requires a large amount of oxidant to achieve radical formation of the substrate. Therefore, most of them need to sacrifice excessive chemical oxidants and stoichiometric metals, which cause environmental pollution and energy consumption, making the large-scale application of this method still limited by sustainability, safety, and cost factors. Therefore, further development to shorten the reaction time, improve the reaction efficiency, and reduce energy consumption in an environmentally friendly, practical, and safe method for CDC will be a continuous process [131–135].

Further progress in this field also needs to identify the radical intermediates and some cationic intermediates involved in the catalytic cycle, for which there is currently still some controversy about the actual reaction pathway. Additional mechanistic and theoretical studies may provide completely new insights into this issue. In addition, CDC reactions involving ether α-C(sp^3^)–H bonds are rarely enantioselective, although examples of enantioselectivity have been reported for some similar CDC reactions of amines, and future developments will undoubtedly bridge this important gap.
